# Adherence to Ebola-specific malaria case management guidelines at health facilities in Guinea during the West African Ebola epidemic

**DOI:** 10.1186/s12936-018-2377-3

**Published:** 2018-06-14

**Authors:** Ian Hennessee, Timothée Guilavogui, Alioune Camara, Eric S. Halsey, Barbara Marston, Deborah McFarland, Matthew Freeman, Mateusz M. Plucinski

**Affiliations:** 1 Division of Parasitic Diseases and Malaria, Centers for Disease Control and Prevention, 1600 Clifton Rd, MS A-04, Atlanta, GA 30306 USA; 2 Rollins School of Public Health, Emory University, Atlanta, GA USA; 3 National Malaria Control Program, Conakry, Guinea; 4 U.S. President’s Malaria Initiative, Centers for Disease Control and Prevention, Atlanta, GA USA

**Keywords:** Malaria, Ebola, Impact, Case management, WHO guidelines

## Abstract

**Background:**

Malaria case management in the context of the 2014–2016 West African Ebola virus disease (EVD) epidemic was complicated by a similar initial clinical presentation of the two diseases. In September 2014, the World Health Organization (WHO) released recommendations titled, “Guidance on temporary malaria control measures in Ebola-affected countries”, which aimed at reducing the risk of EVD transmission and improving malaria outcomes. This guidance recommended malaria diagnostic testing of fever cases only if adequate personal protective equipment (PPE) was available, defined as examination gloves, face shield, disposable gown, boots, and head cover; otherwise presumptive anti-malarial treatment was recommended. The extent to which health workers adhered to these guidelines in affected countries has not been assessed.

**Methods:**

A cross-sectional survey was conducted in 118 health units in Guinea in November 2014 to produce a representative and probabilistic sample of health facilities and patients. Adherence to the EVD-specific malaria case management guidelines during the height of the EVD epidemic was assessed. Associations between case management practices and possible determinants were calculated using multivariate logistic regression, controlling for expected confounders and the complex sample design.

**Results:**

Most (78%) facilities reported availability of examination gloves, but adequate PPE was available at only 27% of facilities. Only 28% of febrile patients received correct malaria case management per the WHO temporary malaria case management guidelines. The most common error was diagnostic testing in the absence of adequate PPE (45% of febrile patients), followed by no presumptive treatment in the absence of adequate PPE (14%). Having had a report of an EVD case at a health facility and health worker-reported participation in EVD-specific malaria trainings were associated with lower odds of diagnostic testing and higher odds of presumptive treatment.

**Conclusions:**

Adherence to guidance on malaria case management in EVD-affected countries was low at the height of the EVD epidemic in Guinea, and there was substantial malaria diagnostic testing in the absence of adequate PPE, which could have contributed to increased EVD transmission in the healthcare setting. Conversely, low presumptive treatment when diagnostic tests were not performed may have led to additional morbidity and mortality among malaria positive patients. National malaria control programs may consider preparing contingency plans for future implementation of temporary changes to malaria case management guidelines to facilitate uptake by health workers. Additional training on standard and transmission-based precautions should help health workers understand how to protect themselves in the face of emerging and unknown pathogens.

## Background

The 2014–2016 West African Ebola virus disease (EVD) led to more than more than 28,000 reported EVD cases and 11,000 EVD-related deaths worldwide—the vast majority of which occurred in Guinea, Liberia, and Sierra Leone. In Guinea alone, there were 3811 reported cases and 2543 deaths [1]. The indirect effects of the outbreak in Guinea and the other affected countries, though difficult to quantify, were profound [2]. Widespread health facility closures and major reductions in treatment-seeking reduced healthcare access [3–5]; this was compounded by reductions in the healthcare workforce due to EVD mortality among health workers [6]. Provision of routine immunization services decreased by as much as 30% [7], resulting in low rates of vaccination coverage and likely contributing to subsequent outbreaks of measles and other vaccine-preventable diseases [8, 9].

Malaria case management posed a particular concern during the EVD epidemic in Guinea, where nationwide malaria prevalence in children under 5 years of age exceeded 40% in 2012 [10]. The EVD epidemic in Guinea accelerated from July to November 2014 and peaked in December 2014 [11]. Most of this time coincided with the highest malaria transmission period in Guinea, which lasts from July to October in most areas of the country [12].

EVD and malaria initially have similar non-specific clinical presentations involving fever, headache, weakness, and joint pain, and may be difficult to distinguish clinically [13]. Confirmation of either disease requires obtaining blood for diagnosis [14–16]. In the absence of adequate PPE, however, diagnostic testing for suspect malaria cases may put health workers at heightened risk of Ebola virus transmission if the patient actually has EVD [13]. Indeed, care for undiagnosed EVD patients is considered a major amplification point for EVD transmission in healthcare settings and likely contributed to the high EVD incidence observed among health workers outside of Ebola treatment units (ETUs) during the epidemic [17, 18]. In 2014, health workers in Guinea had an incidence of EVD infection 42 times higher than the general population [19], and by October 2016 there were 196 confirmed EVD cases and 100 deaths among health workers in Guinea [20].

In September 2014, the World Health Organization (WHO) first circulated temporary malaria case management guidelines for EVD-affected countries, and then issued merged guidance notes in November 2014 [13]. This guidance recommended malaria diagnostic testing of febrile patients in EVD-affected countries only where adequate PPE was available and otherwise recommended presumptive malaria treatment. For suspected malaria patients without vomiting, bleeding, or diarrhoea, adequate PPE was recommended, defined as double examination gloves, face shield (or mask and goggles) and disposable gown. For patients with vomiting, bleeding, or diarrhoea, the guidance recommended full PPE, defined as double examination gloves, impermeable gown (or non-impermeable gown and rubber apron), medical mask, face shield or goggles, head cover, and boots [13]. In addition, the WHO advocated with donors to be flexible with current funding in order to allow affected countries to modify their case management guidelines given the context of the epidemic. Prior to the EVD epidemic, Guinea National Malaria Control Programme (NMCP) and WHO guidelines encouraged diagnostic testing and discouraged presumptive treatment for suspected malaria cases with the 3Ts slogan:test all cases, treat all positive cases, and track each confirmed case through a timely surveillance system. The NMCP had begun scaling up malaria rapid diagnostic test training across the country in the years prior to the epidemic [12, 16, 21].

A cross-sectional survey of health facilities was conducted in December 2014 to examine the effect of the EVD epidemic on malaria case management and treatment seeking in Guinea. This survey showed low rates of diagnostic testing for febrile cases in 2014, which was not accompanied by an increase in presumptive treatment from 2013 to 2014 [21]. This suggested possible confusion among health workers as to whether to test or presumptively treat suspected malaria cases in the context of the EVD epidemic, which could have led to inadequate case management of malaria patients and worse malaria outcomes [22]. However, this study did not directly evaluate the extent to which malaria case management practices by health workers adhered to WHO temporary case management guidelines nor how factors such as trainings and the availability of PPE influenced those practices.

Malaria case management posed an important risk to both patients and health workers during the EVD epidemic in Guinea. However, little information is available on how health workers adhered to EVD-specific malaria case management guidelines or the factors that influenced their decisions. Information is also limited on availability of gloves and other PPE at health facilities, health worker participation in EVD and malaria trainings, and knowledge and attitudes among health workers about malaria case management in the context of the EVD epidemic. This information is needed to inform interventions to improve malaria case management in the context of future EVD or similar epidemics and to reduce risk to health workers and malaria patients. This analysis, therefore, examined adherence to EVD-specific malaria case management guidelines among health workers at health facilities in Guinea and factors associated with those practices. The study was conducted within a study commissioned by the Guinea NMCP that examined the impact of the EVD epidemic on malaria in Guinea in 2014 [21].

## Methods

### Study design and site

**Fig. 1:**
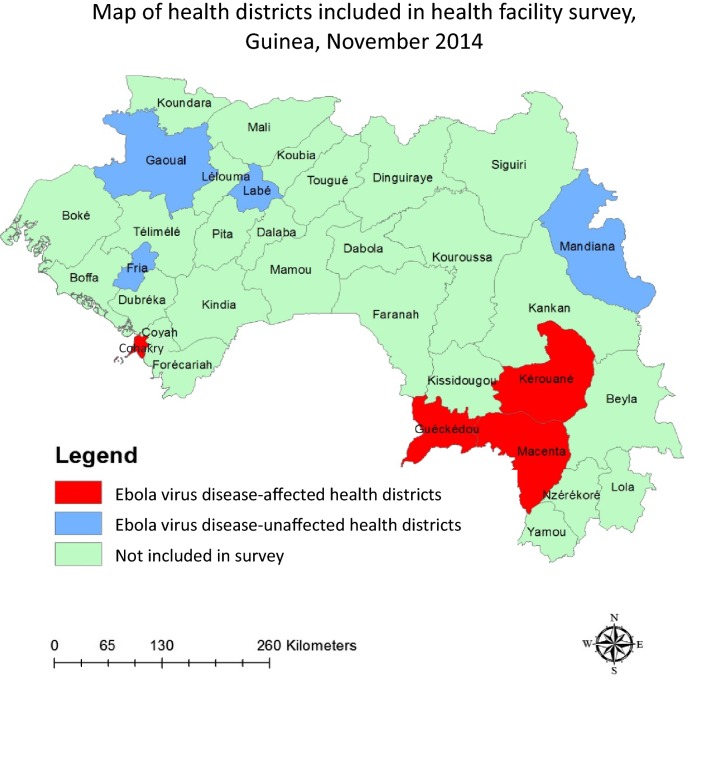
Health districts included in the health facility survey in Guinea, November 2014

A cross-sectional survey of health facilities in eight health districts was conducted in Guinea in December 2014 to produce representative, probabilistic estimates at the health facility and patient level. The four health districts with the highest EVD case counts as of 1 November 2014, were selected: Guéckédou, Kerouané, Macenta, and Conakry. Four health districts that had not confirmed any cases of EVD at the time of the study were also randomly selected, stratified by region; these were Fria, Gaoual, Labé, and Mandiana (Fig. 1). Of note, seven of the health districts included in the study are also prefectures, which is an administrative unit similar to district. Conakry, however, is not a prefecture. Thus the term district is used in this paper.

A total of 120 health facilities were selected in the sample, 60 of which were in EVD-affected districts and 60 of which were in non-affected districts. In each of the eight study districts, 15 health facilities were selected: seven health centres (which serve an average of 10,500 people each) and seven health posts (which serve an average of 3000 people each) were randomly sampled from a list of all health centres and health posts in each district, and the main hospital in each district was automatically included (district hospitals serve an average of 286,000 people per district) [12]. In hospitals with multiple wards, each ward was included as a separate sampling unit. Probability of selection was recorded for each health facility to produce weighted estimates at the health facility level.

### Data collection

Retrospective register abstractions were conducted by trained survey teams at each facility. At each health facility, 20 patient entries were randomly sampled each for the months of November 2014 and November 2013 in order to provide a representative sample of case management practices at each facility during each particular month. Information collected included patient’s age, history of fever, record of malaria diagnostic testing, and record of anti-malarial prescription. The probability of individual record selection based on health facility utilization was recorded in order to produce weighted estimates at the patient level. The sampling strategy is described in further detail elsewhere [21].

Questionnaires on malaria and EVD case management practices were administered to one person in charge at each health post or health centre, typically the head nurse or doctor. At the larger district hospitals, three clinicians were asked to respond to the questionnaire, typically the head doctor and two nurses. Survey questions addressed health worker participation in malaria, EVD, and PPE trainings in the 6 months prior to the survey team visit, health worker reported procedures for handling suspected EVD cases, and reported availability of PPE, malaria diagnostic tests, and anti-malarial drugs at the time of the survey team visits in December 2014. Surveyors obtained verbal consent from all health workers prior to administration of the questionnaire.

### Variable and outcome definitions

Key variables included facility type (health centre, hospital, or health post), rapid diagnostic test (RDT) availability, anti-malarial drug availability, glove availability, availability of adequate PPE, and whether facilities reported having received a case of EVD at their facility (i.e., EVD case report). This definition was solely based on the responses by health workers to survey questions and not confirmed via actual patient records. Because commodity data are not typically recorded on registers at the time of patient visit, the availability of examination gloves was defined as the availability of greater than or equal to 100% of monthly glove consumption average at the particular facility at the time of survey. The availability of RDTs and anti-malarial drugs at the time of patient visit was also defined as greater than or equal to 100% of monthly consumption averages. For patients without vomiting, bleeding, or diarrhoea, adequate PPE availability was defined as health worker-reported availability of double examination gloves, face shield (or mask and goggles) and impermeable gowns at the time of survey. For patients with vomiting, bleeding, or diarrhoea, full PPE availability was defined as health worker-reported availability of double examination gloves, impermeable gown (or non-impermeable gown and rubber apron), medical mask, face shield or goggles, head cover, and boots at the time of survey.

The primary outcomes were malaria diagnostic testing and prescription of anti-malarial drugs without prior diagnostic confirmation (i.e., presumptive treatment). Malaria diagnostic testing was defined as a record of malaria RDT or microscopy. Anti-malarial prescription was defined as the recorded prescription of any type of anti-malarial drug, including artemisinin-based combination therapy (ACT), injectable quinine, or any other unspecified type of anti-malarial drug. Presumptive treatment was defined as the prescription of any type of anti-malarial drug to a febrile patient without prior diagnostic testing by malaria RDT or microscopy.

A third outcome variable, recommended case management, was defined using WHO guidance on temporary malaria control measures in EVD-affected countries to determine whether febrile patients received recommended malaria case management in the context of the EVD epidemic [13]. In accordance with the WHO guidance, recommended case management was defined as (1) conducting a malaria diagnostic test for febrile patients in the presence of adequate PPE *and* treatment according to the result, or (2) presumptive anti-malarial treatment for febrile patients in the absence of adequate PPE. Conversely, non-recommended case management included performing a rapid diagnostic test in the absence of adequate PPE, not conducting a rapid diagnostic test when adequate PPE was present, treatment which did not accord to rapid diagnostic test results, or no presumptive treatment for patients in the absence of adequate PPE. To note, register data did not specify whether the patient had vomiting, diarrhoea, or vomiting; thus the presence of adequate PPE [double examination gloves, face shield (or mask and goggles) and impermeable gown] was considered a minimum prerequisite for performing a malaria rapid diagnostic test. For the purposes of this study, the outcome of recommended case management did not take into account the appropriateness of the malaria drug prescribed or dosing.

### Statistical analysis

Analysis was performed using SAS version 9.3 software (SAS Institute Inc., Cary, North Carolina). Unweighted frequencies were calculated for health facility variables, stratifying by EVD-affected districts versus non-affected districts. Logistic regression models were used to measure differences in each of these characteristics between EVD-affected versus EVD-unaffected districts, adjusting for clustering at the health facility level. Self-reported participation in trainings and knowledge among health workers about malaria and EVD case management were also assessed from health worker questionnaire responses.

Data abstracted from registers from November 2014 were used to calculate descriptive statistics for each outcome of interest. Case management practices for each febrile case were stratified by the availability of adequate PPE to delineate which febrile patients received recommended malaria case management according to the WHO temporary guidelines. Descriptive statistics were then calculated for each outcome of interest, accounting for the complex survey design using sampling weights and cluster and strata statements. Sampling weights were calculated as the product of the inverse of the probability of selection for each facility and the probability of selection of each patient entry. Because there was only one hospital in each district, a strata statement was applied in which each district hospital was assigned its own stratum level. Health posts and health centres were assigned group strata levels. A cluster statement was applied to account for clustering of observations at the health facility level.

Associations were calculated between possible determinants of malaria case management practice and the main outcomes of interest: diagnostic testing, presumptive treatment, and recommended case management. At the district level, location in an EVD-affected district compared to location in an unaffected district was considered as a potential determinant of malaria case management outcomes. At the facility level, variables of interest included EVD case report, reported health worker participation in malaria trainings in the context of the EVD epidemic, EVD trainings, and PPE trainings. Other facility-level variables included availability of examination gloves and adequate PPE, health facility type (hospital, health centre, or health post), and RDT and anti-malarial drug availability. Age category, dichotomized as < 5 or ≥ 5 years old, was evaluated as a possible patient-level determinant of malaria case management practice for febrile patients.

Sampling weights and strata and cluster statements were applied as described above. Multivariable regression models using SAS *surveylogistic* were fitted to assess independent relationships between all measured determinants and malaria case management outcomes, controlling for expected confounders. Backwards model selection was applied to each model using a stay criterion of α < 0.10 in order to produce more parsimonious models. Adjusted odds ratios (aOR) were reported for each independent variable of interest, along with accompanying 95% confidence intervals (95% CI) and 2-sided *p-*values; values less than 0.05 were considered significant.

### Ethical approval

This study consisted of a secondary analysis of de-identified data obtained by the Guinea NMCP during a program evaluation activity and received an exempt research determination from Emory University’s Institutional Review Board. CDC investigators were not considered to be engaged with human subjects (CDC Center for Global Health non-research determination 2015-091).

## Results

### Characteristics of surveyed facilities

Of 120 health facilities in the sampling frame, five facilities in EVD-affected districts were closed due to the EVD epidemic. Two facilities in EVD-unaffected districts were also permanently closed and not included. Because hospitals with multiple wards were included as separate sampling units, 118 units (hereafter referred to as facilities) were included in the sample. A total of 121 health workers were interviewed at open facilities. Monthly supplies of malaria commodities including RDTs and anti-malarial drugs were widely available at > 70% of facilities and did not differ significantly between EVD-affected and EVD-unaffected health districts (Table 1). Adequate PPE was available at 29 (26.6%, 95% confidence interval [CI] 18.3–34.9) of all facilities and full PPE was available at 7 (6.4%, 95% CI 1.8–11.0) facilities. Adequate PPE availability was reported by more health facilities in EVD-affected districts (37.0%, 95% CI 24.2–49.9) than in unaffected districts (16.4%, 95% CI 6.6–26.1, *p *= 0.02). By the end of the study period on 30 Nov 2014, there were no confirmed EVD cases in any of the unaffected districts included in the sample [23]. Health workers in all EVD-affected districts and three of four EVD-unaffected districts nevertheless reported having seen suspected or confirmed EVD case(s) at their facility. Among health workers in EVD-affected districts, 19 (32.8%, 95% CI 20.7–44.8) reported having received at least one EVD case at their facility, whereas 5 (8.5%, 95% CI 1.4–15.6) health workers in EVD-unaffected districts also reported actually having seen at least one case of EVD at their facility. EVD case reports reflected health worker perceptions and were not verified with patient records.

**Table 1:** Characteristics of surveyed health facilities in Guinea, by EVD-affected versus EVD-unaffected district, November 2014

	Total (n = 118)	EVD-affected (n = 55)	EVD-unaffected (n = 58)	*p* value
	n (%, 95% CI)	n (%, 95% CI)	n (%, 95% CI)	
Facility type				
Health centres	47 (39.8, 31.0–48.7)	24 (41.4, 28.7–54.1)	23 (38.3, 26.0–50.6)	0.74
Hospitals	17 (14.4, 8.0–20.7)	11 (19.0, 8.9–29.1)	6 (10.0, 2.4–17.6)	0.18
Health posts	54 (45.8, 36.8–54.8)	23 (39.7, 27.1–52.2)	31 (51.7, 39.0–64.3)	0.20
RDT availability^a^	82 (78.1, 70.2–86.0)	40 (76.9, 65.5–88.4)	42 (79.3, 68.3–90.2)	0.78
Anti-malarial drug availability^a^	78 (72.8, 64.5–81.3)	40 (75.5, 63.9–87.1)	38 (70.4, 58.2–82.6)	0.56
PPE availability				
Gloves^a^	82 (78.1, 70.2–86.0)	39 (75.0, 63.2–86.8)	43 (81.1, 70.6–91.7)	0.45
Adequate PPE^b^	29 (26.6, 18.3–34.9)	20 (37.0, 24.2–49.9)	9 (16.4, 6.6–26.1)	0.02
Full PPE^c^	7 (6.4, 1.8–11.0)	6 (11.1, 2.7–19.5)	1 (1.8, 0.0–5.4)	0.09
Health workers reporting EVD case(s)	24 (20.5, 13.2–27.8)	19 (32.8, 20.7–44.8)	5 (8.5, 1.4–15.6)	0.0024

Results are unweighted; *RDT* rapid diagnostic test, *PPE* personal protective equipment, *95% CI* confidence interval

^a^≥ 100% of monthly commodity consumption average available at time of survey

^b^Reported availability of examination gloves, face shield, disposable gown, boots, and head cover on day of survey visit

^c^Reported availability of examination gloves, face shield, impermeable gown (or non-impermeable gown and rubber apron), medical mask, head cover, and boots on day of survey visit

### Health worker training and knowledge about EVD and malaria case management in context of EVD epidemic

More than 70% of surveyed health workers reported having received some type of information on EVD identification and case management in the 6 months before the survey (Table 2). Among those who reported having received any EVD information, more health workers in EVD-affected districts had received specific EVD training (63.3%, 95% CI 38.6–88.0) compared to unaffected districts (30.0%, 95% CI 10.4–49.6, *p *= 0.04). Training and posters were the most common sources of EVD-related information among health workers in EVD-affected districts, whereas training and radio were the most common sources in EVD-unaffected districts. Fewer health workers (35.1%, 95% CI 21.5–48.6) reported having received any information on malaria case management in the context of the EVD epidemic. Among health workers who reported receiving information about malaria case management, almost 70% (95% CI 20.8–100.0) in EVD-affected districts reported receiving specific trainings on the subject, compared to 32.5% (95% CI 2.4–62.6) in EVD-unaffected districts (*p *= 0.20).

**Table 2:** Reported trainings and knowledge among health workers about EVD and malaria case management in the context of the EVD epidemic, Guinea 2014

	Total (n = 121)	EVD-affected (n = 60)	EVD-unaffected (n = 61)	*p* value
	% (95% CI)	% (95% CI)	% (95% CI)	
Received any information on EVD identification and case management	72.9 (61.0–84.8)	68.6 (48.8–88.3)	78.0 (66.2–89.8)	0.39
Training^a^	46.9 (30.9–62.8)	63.3 (38.6–88.0)	30.0 (10.4–49.6)	0.04
Supervisor	13.9 (0.9–26.8)	18.5 (0.0–39.9)	9.1 (0.0–24.7)	0.47
Internet	0.7 (0.0–2.24)	0.0 (0.0–0.0)	1.5 (0.0–4.6)	< 0.001
Radio	33.3 (16.8–49.8)	26.6 (0.3–52.8)	40.2 (17.7–62.7)	0.43
Poster	19.9 (3.9–36.0)	37.9 (11.2–64.6)	1.4 (0.0–3.2)	< 0.001
Received any information on malaria case management in the context of the EVD epidemic	35.1 (21.5–48.6)	32.2 (11.6–52.7)	38.4 (20.7–56.2)	0.64
Training^a^	50.9 (25.1–76.8)	69.7 (20.8–100.0)	32.5 (2.4–62.6)	0.20
Supervisor	11.0 (0.0–27.1)	5.7 (0.2–11.3)	16.2 (0.0–47.7)	0.32
Internet	0.0 (0.0–0.0)	0.0 (0.0–0.0)	0.0 (0.0–0.0)	–
Radio	19.6 (0.0–46.9)	29.7 (0.0–78.9)	9.8 (0.0–30.5)	0.37
Poster	19.0 (0.0–45.6)	38.3 (0.0–85.0)	0.0 (0.0–0.0)	–
Received PPE training in previous 6 months	59.5 (45.8–73.2)	80.0 (60.6–99.4)	35.5 (19.3–51.7)	0.0041
Knows how to correctly identify suspected EVD case (self-report)	93.0 (87.9–98.0)	98.7 (96.8–100.0)	86.3 (76.0–96.6)	0.0036
Correctly identified ≥ 5 clinical symptoms of a suspected EVD case	65.3 (51.5–79.1)	84.0 (68.4–99.6)	40.3 (21.6–59.0)	0.0029
Differentiate between suspected EVD case and suspected malaria case by				
Malaria rapid diagnostic test	49.8 (35.4–64.1)	36.0 (16.6–55.4)	68.2 (47.8–88.6)	0.03
Notion of contact with EVD case	34.5 (20.2–48.8)	41.8 (21.0–62.5)	24.7 (4.4–45.1)	0.25
Symptoms	79.5 (68.5–90.6)	85.8 (75.3–96.3)	71.2 (50.7–91.6)	0.16

Note: Results are weighted

*EVD* Ebola virus disease, *PPE* personal protective equipment, *95% CI* confidence interval

^a^Sources from which health workers reported receiving information on EVD identification and case management/malaria case management in the context of the EVD epidemic

A high proportion of health workers in both EVD-affected (98.7%, 95% CI 96.8–100.0) and EVD-unaffected (86.3%, 95% CI 76.0–96.6) districts self-reported being able to correctly identify a suspected EVD case. A similarly high proportion of health workers in EVD affected districts (84.0%, 95% CI 68.4–99.6) were able to correctly identify at least five clinical symptoms of suspect EVD cases, whereas 40.3% (95% CI 21.6–59.0) of health workers in EVD-unaffected districts could do the same (*p *=0.0029). When asked about how to differentiate between suspected EVD and malaria cases, health workers most frequently reported clinical symptoms as a means of differentiation (79.5%, 95% CI 68.5–90.6). Relatively few (36.0%, 95% CI 16.6–55.5) health workers in EVD-affected districts suggested using malaria rapid diagnostic tests as a means of differentiation, compared to 68.2% (95% CI 47.8–88.6) in EVD-unaffected districts (*p *= 0.03).

### Malaria case management characteristics at surveyed health facilities

A total of 4963 patient records were abstracted from health facility registers from November 2014. Among 4019 patient records where history of fever was recorded, 2502 (62.2%) presented with fever.

**Fig. 2:**
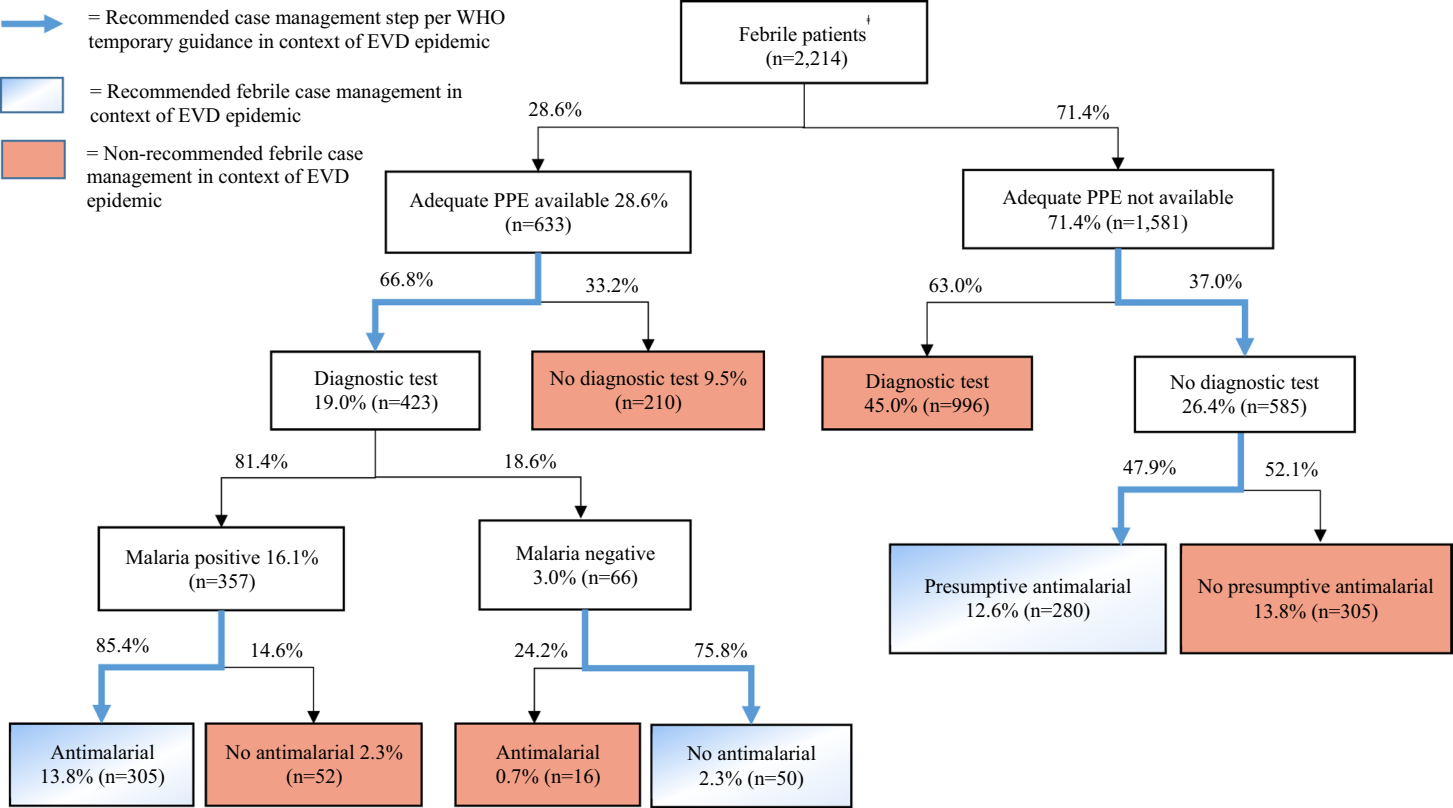
Adherence to Ebola-specific malaria case management guidelines for febrile patients at health facilities in Guinea in November 2014. Percentages shown in boxes represent percent of total febrile patients, whereas percentages shown outside of boxes represent the percent of each subsection. ^ǂ^Only includes febrile patients for whom health facility PPE information was available

Adequate PPE was available for 633 (28.6%) of the 2214 febrile patients for whom health facility PPE information was available (Fig. 2). Of these, 423 (66.8%) received a malaria diagnostic test, whereas 210 (33.2%) did not. Among malaria positive cases, 305 (85.4%) received an anti-malarial prescription. Among the 66 cases that had a negative malaria diagnostic test result, 16 (24.2%) nevertheless received an anti-malarial prescription.

Adequate PPE was not available for 1581 febrile patients (71.4%). Despite WHO guidance that recommended presumptive anti-malarial treatment for suspected malaria cases in the absence of adequate PPE, 996 (63%) of febrile patients for whom no adequate PPE was available still received a malaria diagnostic test. Overall, 45.0% of all febrile patients received a diagnostic test at health facilities without a sufficient supply of adequate PPE. Of the 585 (37%) patients for whom adequate PPE was not available and who did not receive a diagnostic test, 280 (47.9%) were presumptively prescribed an anti-malarial.

Adjusting for sampling weights, a total of 59.5% of patients presented with fever at health facilities in Guinea in November 2014 (Table 3). Among febrile patients, 62.1% (95% CI 52.6–71.6) received a malaria diagnostic test, and 73.2% (95% CI 67.9–78.4) of diagnostic tests were positive. The proportion of febrile patients treated according to the test result was 72.3% (95% CI 58.9–85.7). Among patients who had a positive malaria diagnostic test, 86.1% (95% CI 77.1–95.1) were treated with anti-malarial drugs. A substantial proportion of patients with negative diagnostic tests were also treated with anti-malarial drugs (40.0%, 95% CI 29.5–50.5).

**Table 3:** Case management characteristics of patients at health facilities in Guinea, November 2014

	n = 4963
	% (95% CI)
Patients with fever	59.5 (53.4–65.6)
Diagnostic test for febrile patients	62.1 (52.6–71.6)
Test positivity	73.2 (67.9–78.4)
Treated according to result	72.3 (58.9–85.7)
No diagnostic test for febrile patients	37.9 (28.4–47.4)
Presumptive treatment	48.5 (36.7–60.4)
Recommended febrile case management^a^	27.8 (18.9–36.8)

Results are weighted; *95% CI* confidence interval

^a^Recommended febrile case management defined as: diagnostic test for febrile patients in presence of adequate personal protective equipment (PPE) and treatment according to result *or* presumptive anti-malarial treatment for febrile patients in absence of adequate PPE

Among the febrile patients who did not receive a diagnostic test, 48.5% (95% CI 36.7–60.4) received presumptive treatment. Overall, 27.8% (95% CI 18.9–36.8) of febrile patients received recommended malaria case management, either being administered a malaria diagnostic test in the presence of adequate PPE *and* being treated according to the result *or* receiving presumptive anti-malarial prescription in the absence of adequate PPE.

Overall, 72.5% of diagnostic tests across all facilities were conducted at facilities with sufficient supply of gloves. A substantially lower proportion of diagnostic tests (24.9%) were conducted at facilities with sufficient supply of adequate PPE, and 26.5% of presumptive treatment was at facilities with adequate PPE.

### Factors associated with malaria case management practices

**Fig. 3:**
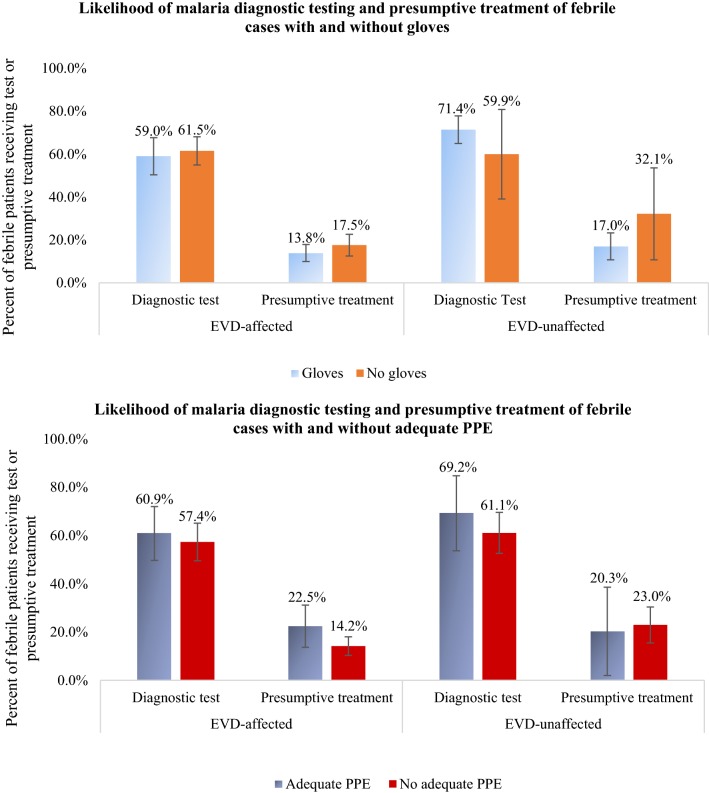
Malaria diagnostic testing and presumptive treatment for febrile cases with gloves and adequate personal protective equipment (PPE), stratified by EVD-affected health district, Guinea, November 2014

Prior to controlling for other variables, the presence of gloves or adequate PPE did not appear to influence the probability of diagnostic testing and presumptive treatment for febrile patients in either EVD-affected or EVD-unaffected districts (Fig. 3). Rates of diagnostic testing and presumptive treatment were similar for patients in facilities with and without gloves, both in EVD-affected and EVD-unaffected districts. Similar trends were observed for rates of diagnostic testing and presumptive treatment for facilities with and without adequate PPE in EVD-affected and EVD-unaffected districts.

In the fully adjusted model, receiving care at a facility where health workers reported having received a previous EVD case was associated with dramatically lower likelihood of diagnostic testing compared to being cared for at a facility that had not reported having received a suspected EVD case [adjusted odds ratio (aOR) = 0.04, 95% CI 0.01, 0.18], as was reported participation in malaria trainings (aOR = 0.21, 95% CI 0.05, 0.88) (Table 4). Glove availability, on the other hand, was associated with higher likelihood of diagnostic testing, as was health worker reported participation in PPE trainings and anti-malarial drug availability. Health centres also appeared more likely to provide diagnostic tests for febrile patients compared to hospitals and health posts, controlling for all other factors.

**Table 4:** Factors associated with malaria case management practices for febrile patients at health facilities in Guinea, November 2014 (N = 2502)

	Diagnostic testing		Presumptive treatment		Recommended febrile case management^e^	
	aOR	95% CI	aOR	95% CI	aOR	95% CI
District-level						
EVD-affected district	–	–	0.07*	0.01–0.73	–	–
Facility/health worker-level						
EVD case report	0.04*	0.01–0.18	982*	24.9–999	–	–
Glove availability^a^	5.38*	1.96–14.7	0.21*	0.06–0.74	–	–
Adequate PPE availability^b^	–	–	4.75	0.83–27.1	192*	12.1–> 999
Malaria training^c^	0.21*	0.05–0.88	12.2*	1.20–124	2.97	0.91–9.73
EVD training^d^	–	–	–	–	0.25	0.05–1.14
Received PPE training	4.97*	1.78–13.8	–	–		
Anti-malarial availability^a^	10.4*	4.29–25.1	0.06*	0.01–0.31	0.08*	0.01–0.69
RDT availability^a^	–	–			19.3	0.81–462
Facility						
Health centre	3.55*	1.03–12.2	0.09*	0.02–0.36	0.04*	0.01–0.37
Hospital	–	–	< 0.01*	< 0.01–0.02	0.02*	< 0.01–0.36
Health post (ref)	1	–	–	–	–	–
Patient-level						
Age ≥ 5 years old	–	–	0.54*	0.36–0.79	0.43*	0.21–0.88

*RDT* rapid diagnostic test, *PPE* personal protective equipment, *EVD* Ebola virus disease, *95% CI* confidence interval

* Significant at *p *< 0.05 Note: results are weighted

– Signifies variable that was included in full model but fell out of model during backwards model selection using stay criterion of α < 0.10

^a^Availability: Availability of examination gloves defined as ≥ 100% of average monthly glove consumption available at time of survey visit

^b^Reported availability of gloves, apron, boots, helmet, and face screen at time of survey visit

^c^Health worker reported having received training on malaria case management in context of EVD epidemic in previous 6 months

^d^Health worker reported having received training on EVD case management and identification in context of EVD epidemic in previous 6 months

^e^Recommended febrile case management defined as diagnostic test for febrile patients in presence of adequate PPE and treatment according to result *or* presumptive anti-malarial treatment for febrile patients in absence of adequate PPE

By contrast, EVD case report was associated with higher likelihood of presumptive treatment (aOR = 982, 95% CI 24.9, 999), as was participation in malaria trainings (aOR = 12.2, 95% CI 1.20, 124). Location in an EVD-affected district, however, was associated with lower odds of presumptive treatment (aOR = 0.07, 95% CI 0.01, 0.73). Glove availability was associated with lower odds of presumptive treatment, as was anti-malarial availability. Health centres and hospitals both appeared less likely to presumptively treat than health posts, and patients 5 years old and older were less likely to be presumptively treated than those under 5 years.

Adequate PPE availability was associated with higher likelihood of recommended case management per WHO temporary guidelines (aOR = 192, 95% CI 12.1, > 999). Anti-malarial availability was associated with lower odds of recommended case management, as was patient age of 5 years and older compared to under 5 years. Health centres and hospitals were both associated with lower odds of recommended case management compared to health posts.

### EVD case management practices at survey health facilities

**Fig. 4:**
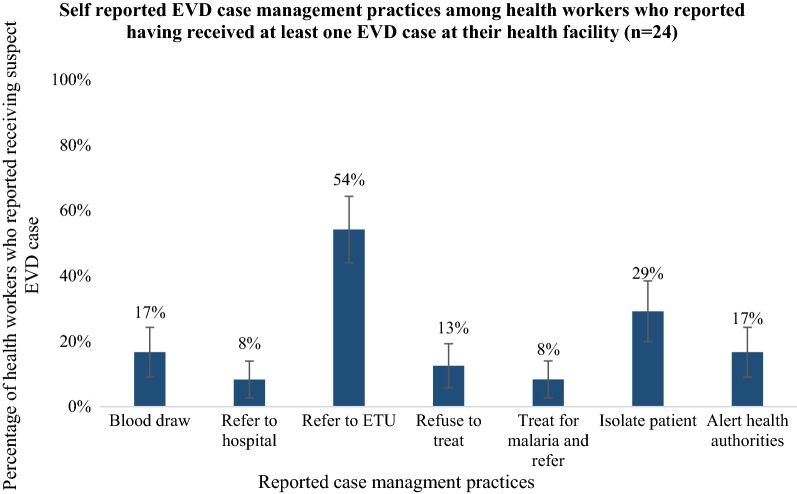
Self-reported Ebola-virus disease (EVD) case management practices among health workers who reported having received at least one EVD case at their health facility (n = 24), Guinea, 2014. Respondents could choose more than one reply

Health workers who reported having received a suspected EVD case at their facility (n = 24) were asked what case management actions they took for that patient (Fig. 4). The highest proportion of respondents (54.2%) reported referring the suspected case to an ETU, whereas the next most common response was isolating the patient (29.2%). Of the 16.7% of respondents who reported drawing blood from the suspected EVD patient, 75% had adequate PPE at their facility, but none of them had full PPE available at their facility. A small proportion (8.3%) of respondents reported treating suspected EVD patients for malaria before referring them to an ETU or other health facility.

## Discussion

This study examined adherence to EVD-specific malaria case management guidelines by health workers at health facilities in Guinea in the context of the EVD-epidemic, and factors associated with those practices. Less than 30% of all febrile patients received recommended malaria case management per WHO temporary guidance, which could have led to increased EVD infection risk among health workers and worse outcomes among malaria patients. Among patients who were tested for malaria, more than three-fourths of these tests were conducted at health facilities without sufficient supply of adequate PPE.

Fewer than 50% of patients who did not receive a malaria diagnostic test received presumptive malaria treatment, whereas many of the patients who did receive malaria diagnostic tests could have benefited from presumptive treatment instead when adequate PPE was not available. Because malaria prevalence in children under 5 years of age can exceed 40% in Guinea [10], a substantial number of malaria episodes were likely left untreated due to low presumptive treatment for patients who did not receive a diagnostic test. If the patients did not seek care elsewhere, their risk for severe disease and death would have been elevated [22, 24]. Increased malaria morbidity may have resulted in additional treatment seeking, which would have added additional burden to a health system already in crisis. Additionally, the proportion of patients who were treated according to their malaria diagnostic test was inadequate at 72%. This likely resulted in additional malaria morbidity for patients with malaria who did not receive an anti-malarial drug [25].

Non-recommended malaria case management may have put health workers at higher risk for EVD infection during the EVD epidemic. While glove availability was above 75% at facilities in both EVD-affected and EVD-unaffected districts, less than one-third of facilities had sufficient supplies of adequate PPE and only 6% reported full PPE availability. The majority of malaria diagnostic tests were conducted without sufficient supply of adequate PPE, which could have put health workers at elevated risk of EVD infection if a patient had EVD [6]. Glove availability was associated with an increased likelihood of diagnostic testing for febrile patients and decreased likelihood of presumptive treatment. While this may have been protective for health workers in the event that suspected malaria patients had undiagnosed EVD, gloves alone were not recommended because they provide inadequate protection against Ebola virus infection [13].

Reported participation in EVD, malaria, and PPE trainings and knowledge of EVD and malaria case management practices were higher among health workers in EVD-affected districts, an indication that response efforts were targeted to EVD-affected districts. Reported participation in malaria and PPE trainings in the context of the EVD epidemic was associated with a lower likelihood of diagnostic testing for febrile patients, and malaria trainings were associated with higher likelihood of presumptive anti-malarial treatment. This suggests that trainings successfully encouraged safer malaria case management practices by health workers during the EVD epidemic.

The observed strong association between EVD case report and malaria case management practices suggests that first-hand contact with an EVD patient, whether confirmed or suspected, had an important influence on the ways that health workers managed suspected malaria cases during the EVD epidemic. After health facilities had received a suspected EVD patient, health workers at that facility may have been more wary of close patient contact and may have been more likely to follow the temporary guidance to presumptively treat suspected malaria cases rather than perform malaria diagnostic tests. This may have had a protective effect for those health workers because, in the absence of adequate PPE, rapid diagnostic testing for febrile patients may increase EVD transmission risk [13].

In Liberia in 2015, an infection prevention and control (IPC) intervention called ‘ring IPC’ was implemented in which surrounding high risk health facilities received intensive PPE and IPC training and support immediately following the detection of a local EVD case. This method appeared effective in increasing health workers’ ability to identify and isolate suspected EVD patients, which likely reduced health worker infection and onward EVD transmission [26]. A similar approach could be considered for training or reinforcing revised malaria case management guidelines in high-risk facilities immediately after an EVD case is detected locally. Because this localized training approach is immediately relevant for at-risk health workers, it may be effective in promoting behavior change and safe case management practices.

Certain EVD case management practices by health workers who reported having received at least one EVD case at their facility may have increased risk for those health workers and malaria patients. Among the health workers who reported performing a blood draw for suspected EVD cases, the lack of full PPE available at their facility may have constituted a high risk for EVD transmission [13, 19]. Additionally, because malaria-Ebola virus coinfection was common in Guinea [27] and less than 10% of health workers reported presumptively treating suspected EVD cases for malaria prior to referring to an ETU, untreated malaria in patients with EVD may have increased their risk of mortality [28]. The WHO temporary malaria case management guidance did not specifically include information on malaria case management practices for suspected EVD cases. Such information could have reduced confusion among health workers on whether suspected EVD cases should be presumptively treated for malaria or if and when an RDT should be performed.

Among the limitations of this study, the reliance on proxy measurements for the availability of PPE, RDTs, and anti-malarial drugs at the time of patient visit might have distorted the true influence that the availability of those commodities likely had on health worker practices. Also, because the study was unable to assess whether the patient had vomiting, diarrhoea or bleeding at the time of visit, a determination could not be made of which diagnostic tests should have been conducted with full PPE instead of adequate PPE according to the WHO temporary guidance. This may have led to an underestimation of the proportion of febrile patients who received non-recommended malaria case management in the context of the EVD epidemic. Additionally, the register abstraction only recorded ‘other anti-malarial’ if a patient received an anti-malarial prescription other than ACT or injectable quinine. This may have led to overestimation of the proportion of malaria patients who received recommended malaria case management, as some of them may have received non-recommended anti-malarial treatment. Finally, the absence of outcome data in patient registers limited the ability of this study to directly assess the effect of case management practices on malaria morbidity and mortality.

## Conclusion

Many malaria case management practices at health facilities in Guinea were not in line with WHO temporary guidance introduced during the EVD epidemic, with a high proportion of diagnostic tests performed without the presence of adequate PPE and low rates of presumptive treatment for febrile patients who did not receive a diagnostic test. This may have contributed to excess morbidity and mortality among malaria patients and increased risk of Ebola virus infection in health workers. Malaria and PPE trainings in the context of the EVD epidemic appeared successful in reducing the likelihood of diagnostic testing in the absence of adequate PPE and increasing the likelihood of presumptive treatment, though this was not as complete as hoped for.

In the event of another EVD epidemic or similar outbreak, the Guinea Ministry of Health and NMCP may consider preparing contingency plans for rapid dissemination and implementation of temporary changes to malaria case management guidelines. Response efforts should ensure health workers are aware of and practicing current case management guidelines, and adequate PPE is available at all facilities. Additionally, basic training on standard and transmission-based precautions may help health workers understand how to protect themselves in the face of emerging and unknown pathogens.
